# Double-Grating Displacement Structure for Improving the Light Extraction Efficiency of LEDs

**DOI:** 10.1100/2012/515468

**Published:** 2012-10-15

**Authors:** Zhibin Wang, Yang Hao, Zhongdong Wang, Xian Liu, Qian Zhang, Dandan Zhu

**Affiliations:** ^1^College of Electrical Engineering, Yanshan University, Qinhuangdao 066004, China; ^2^Mechanical Science and Engineering Institute, Northeast Petroleum University, Daqing 163318, China

## Abstract

To improve the light extraction efficiency of light-emitting diodes (LEDs), grating patterns were etched on GaN and silver film surfaces. The grating-patterned surface etching enabled the establishment of an LED model with a double-grating displacement structure that is based on the surface plasmon resonance principle. A numerical simulation was conducted using the finite difference time domain method. The influence of different grating periods for GaN surface and silver film thickness on light extraction efficiency was analyzed. The light extraction efficiency of LEDs was highest when the grating period satisfied grating coupling conditions. The wavelength of the highest value was also close to the light wavelength of the medium. The plasmon resonance frequencies on both sides of the silver film were affected by silver film thickness. With increasing film thickness, plasmon resonance frequency tended toward the same value and light extraction efficiency reached its maximum. When the grating period for the GaN surface was 365 nm and the silver film thickness was 390 nm, light extraction efficiency reached a maximum of 55%.

## 1. Introduction

Low-carbon economy and green economy have recently become major themes of global development. As a type of green lighting energy, light-emitting diodes (LEDs) have attracted considerable research attention [1]. LEDs present the advantages of energy conservation, environment friendliness, fast response, and long lifetimes. Thus, LEDs lighting systems have been considered to reduce greenhouse gas emissions by replacing fuel-based lighting in the developing world [2]. Not only that, these light sources are extensively used as backlight sources of liquid crystal displays, advertisements, night lights, traffic signals, outdoor lighting, full color displays [3], and biometric devices [4], in addition, the array of LEDs has been used to inactivate bacteria in liquid suspension and on exposed surfaces [5], among other applications. Despite these advantages, the development of LED core technology is constrained by the photons produced in the lighting devices. Such a constraint prevents photons from being effectively radiated and converted into available optical power. This problem stems from the inherent structure of solid-state lighting devices: the refractive index of solid semiconductor luminophore is higher than that of the surrounding medium [6]. Thus, the light emitted by the lighting devices easily causes total internal reflection in the interface of semiconductor and air, after which the light retraces the optical path of the luminophore and is converted into heat [7]. This phenomenon makes the LEDs working in high-temperature environments for a long time; it not only wastes energy, but also shortens LED lifetimes. So the improvement of LEDs light extraction efficiency can not only extend lifetime, but also make important contribution to the development of the new green energy.

Foreign and domestic experts have begun improving the structure of LED chips, thereby significantly enhancing light extraction efficiency. These improvements primarily include changing the geometric shape of the chips, reducing the duration of total internal reflection in the chips, and decreasing light energy losses. The methods generally applied in realizing such enhancements are using the inverted pyramid structure [8, 9], performing surface roughening [10], constructing a mirror with Bragg grating reflection [11], and creating a two-dimensional photonic crystal structure on the surface of GaN [12–15]. Using the characteristic of surface plasmons (SPs) to enhance the light extraction efficiency of LEDs has recently become a research focus [16]. Compare with a single grating structure of the LED model described in [17]. In the current work, a double-grating displacement structure was constructed for an LED model to improve the light extraction efficiency of LEDs. The structure was realized by etching grating patterns on GaN and metallic silver film surfaces.

Under certain conditions, the free electrons of metal surfaces produce collective coherent oscillation, which is stimulated by light; the electromagnetic wave at the metal/air interface is called SP [18, 19]. The internal quantum efficiency and external quantum efficiency of LEDs can be improved by using SPs. The improvement in the internal quantum efficiency of LEDs by applying SPs is based on a principle that is related to the spontaneous radiative rate of excitons and state density [20]. When the light center is located in the micro cavity of the wavelength scale, the state density of photons changes, which, in turn, causes variations in the spontaneous radiation rate of excitons. The principle governing the use of SPs to improve the external quantum efficiency of LEDs is based on the observation that light can excite SPs. The light reflection angle is greater than the total internal reflection angle, but the former prevents light from radiating outward. A reasonable metal structure can excite SPs, which are then radiated outward in the form of light. Part of the SPs can be coupled to the electromagnetic waves on the surface of metal, and radiated outward in the form of light by etching a grating-coupled structure on the surface. Part of the incident light that causes total internal reflection can be diminished by grating-patterned etching of the GaN surface. Reducing total internal reflection can also be realized by using a dielectric material with a high coefficient. Using this material produces an evanescent field in total internal reflection. The evanescent field is used as the excitation source that stimulates the SP resonance. It can effectively extract a limited amount of light and reduce the junction temperature of LED chips. Compared with the traditional LED structure, the grating etched onto the GaN surface is relatively complex when used without system optimization. In this work, therefore, the grating period for GaN and metallic silver film thickness was optimized. The influence of these parameters on light extraction efficiency was also simulated and analyzed.

## 2. Theories and Methods

### 2.1. Introduction to SP Resonance

When the wave frequency for the excitation of SPs is larger than the plasmon resonance frequency (*ω*_*p*_), the electromagnetic field is radiatively propagated far from the space between the metal/dielectric material interface, a phenomenon described as the radiative mode of SPs [20, 21]. Only when the horizontal direction wave vector **k**_*x*_ is very small (i.e., resonance frequency ≈*ω*_*p*_) can the life cycle of the radiant mode of SPs on a metal surface be defined. The gradual increase in **k**_*x*_ results in a resonance mode life cycle that is too short to have any practical significance. The SP resonance in the range of visible frequency is the nonradiative SP mode. The electromagnetic field produced by this mode is limited to an area near the metal surface. This phenomenon is called electromagnetic wave dispersion.

**Figure 1:**
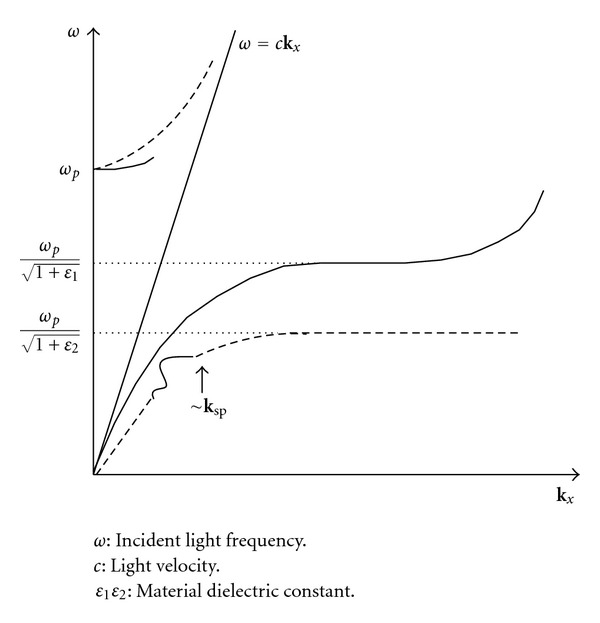
Dispersion relationship curve of SPs.

**Figure 2:**
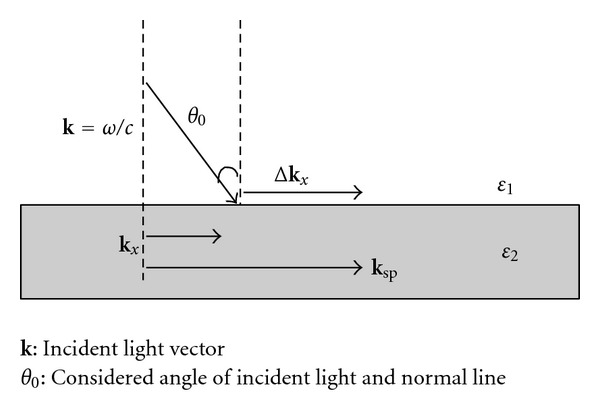
SPs excited by light.

The SP dispersion relationship is depicted in Figure 1. The dispersion curve of the nonradiative SPs produced on a metal surface lies completely on the right side of the incident electromagnetic wave line of the dielectric material [20]. This result indicates that the SPs have a longer horizontal component of wave vector **k**_sp_ than that of the incident electromagnetic wave vector **k**_*x*_ of the same energy. Thus, the general electromagnetic waves incident from the dielectric material cannot stimulate nonradiative SPs and reach the resonance mode. To match the excitation conditions for the SP resonance mode, a coupled mechanism should be applied. This mechanism increases wave vector Δ**k**_*x*_ and causes the incident electromagnetic wave to achieve a higher **k**_*x*_ (Figure 2).

Two coupled mechanisms are commonly used [20]. One involves producing a small-raster periodic structure on a metal surface as a coupled medium. Under an external electromagnetic field, the free electrons of the metal surface in these cycle structures cause polarized electron oscillation for a specific wavelength. The generated electromagnetic field can provide an additional value Δ**k**_*x*_ to the incident electro-magnetic wave. Subsequently, SP resonance is stimulated. The other coupled mechanism uses the total internal reflection dissipation field produced in a material with a high dielectric constant as the excitation source to stimulate SP resonance. 

The electromagnetic wave vector can be written as

(1) $$\begin{array}{c} k^{2}=\frac{\omega^{2}\varepsilon \mu }{c^{2}}, \end{array}$$

where *ε* and *μ* are the relative permittivity and relative permeability of the material, respectively. When the electromagnetic wave passes through the prism of high permittivity, the wave vector is greater than that observed in dielectric material *ε*_1_. When the incident light produces total internal reflection in the prism/dielectric material interface, part of the evanescent field is tunneled into the dielectric material near the total internal reflection interface. The wave vector **k**_*x*_ of the evanescent field and the total internal reflection wave vector have the same size when the distance between the prism and the metal surface is sufficiently small. At the same time, when the incident light wave vector horizontal component of total internal reflection satisfies the conditions of SPs, the SPs in the dielectric material/metal interface are stimulated.

### 2.2. Finite Difference Time Domain Method

The finite difference time domain (FDTD) method [22], an extension of the finite difference method, is a numerical analysis approach that directly performs computer simulations by using Maxwell equations for electromagnetic fields. Using the Maxwell curl equations in accordance with the field quantities that have special configurations in the Yee grid yields [23, 24]

(2) ε(i+1/2,j,k)[Exn+1(i+1/2,j,k)−Exn(i+1/2,j,k)]Δt +σ(i+1/2,j,k)[Exn+1(i+1/2,j,k)+Exn(i+1/2,j,k)]2  =[Hzn+1/2(i+1/2,j+1/2,k)−Hzn+1/2(i+1/2,j−1/2,k)]Δy   −[Hyn+1/2(i+1/2,j,k+1/2)−Hyn+1/2(i+1/2,j,k−1/2)]Δz.

Let

(3) $$\begin{array}{c} p=\left(i+\frac{1}{2},j,k\right),\;\;q^{+}=\left(i+\frac{1}{2},j+\frac{1}{2},k\right),\\ q^{-}=\left(i+\frac{1}{2},j-\frac{1}{2},k\right),\\ r^{+}=\left(i+\frac{1}{2},j,k+\frac{1}{2}\right),\;\;r^{-}=\left(i+\frac{1}{2},j,k-\frac{1}{2}\right). \end{array}$$

Thus,

(4) (Ex)pn+1=[1−Δtσp/2εp](Ex)pn[1+Δtσp/2εp]+(Δt/εp){[(Hz)q+n+1/2−(Hz)q−n+1/2]/Δy−[(Hy)r+n+1/2−(Hy)r−n+1/2]/Δz}[1+Δtσp/2εp],

where the superscripts *n* + 1/2 and *n* are the time step-numbers; a group of changes (*i*,*j*,*k*) and Δ*t* are the time steps; Δ*x*,  Δ*y*,  Δ*z* are the distances of the adjacent lattice point in the *x*,  *y*,  *z* directions, respectively. Other field quantities can be treated in the same manner.

To perform a stable numerical simulation, the variables should satisfy

(5) $$\begin{array}{c} v_{\max}\Delta t\leq \frac{1}{\sqrt{{\left(1/\Delta x\right)}^{2}+{\left(1/\Delta y\right)}^{2}+{\left(1/\Delta z\right)}^{2}}}, \end{array}$$

where *v*_max⁡_ is the maximum phase velocity, Δ*t* denotes the time step, and Δ*x*,  Δ*y*,  Δ*z* are the distance steps in the *x*,  *y*,  *z* directions, respectively. When Δ*x* = Δ*y* = Δ*z* = Δ, (5) can be simplified as $v_{\max}\Delta t<\Delta /\sqrt{3}$. To reduce the error caused by numerical dispersion effects, (5) should also satisfy the conditions Δ/*λ*_min⁡_ < 1/10, where *λ*_min⁡_ is considered the shortest wavelength of the electromagnetic wave considered.

The FDTD method was used to solve the numerical analysis problems presented by grating-patterned etching on the metallic silver film. This approach also enables the excitation of SPs and application of the total internal reflection dissipation field for exciting the SPs. The changes in time step Δ*t* reflect the variations in energy flow on the receiving surface. This reflection is realized by placing a receiving surface over the structure. The changes in energy flow facilitate the analysis of the effects of structural parameter variations on the light extraction efficiency of LEDs.

## 3. Physical LED Model

**Figure 3:**
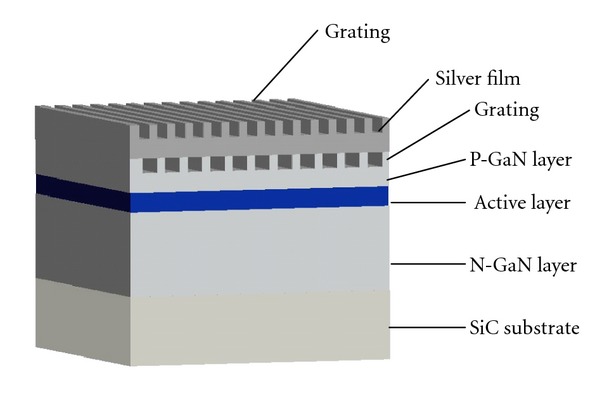
Physical model of an LED with a double-grating displacement structure.

The physical model of the LED with a double-grating displacement structure was constructed by grating-patterned etching on the GaN and metallic silver film surfaces (Figure 3). The model comprises the silver film layer, P-GaN layer, active layer, N-GaN layer, and SiC substrate. During the experiment, the calculated area dimensions of 4200 nm × 420 nm were realized; the thicknesses of the P-GaN and N-GaN layers were 200 and 400 nm, respectively. Compare with the physical LED model as described in [17], a grating-patterned etching was added to the GaN surface, which had two reasons. (1) Grating-patterned etching can diminish part of the total internal reflection produced by incident light. (2) A portion of the light causes the evanescent field of total internal reflection to excite SPs; The study in [7] has discussed that grating-patterned etching on the top surface can effectively enhance the light extraction efficiency of LEDs; thus, this phenomenon results in a combined effect with grating-patterned etching on the metallic silver film surface, thereby enhancing optical transmission and improving the light extraction efficiency of LEDs.

## 4. Numerical Simulation and Analysis

A numerical simulation was conducted using the FDTD method. The wavelength of light was 500 nm, the refractive index of air was 1.0, and the refractive index of GaN was 2.4. The silver film was used in the Lorentz dispersion model. In analyzing the light absorption process, the silver film is disregarded. The light emitted by the active layer is replaced by the total field-scattered field source, an approach that realizes incident plane waves from all directions. The intensity was 1 and the grating period of the metallic silver film was 375 nm. A receiving surface, which is capable of reflecting light intensity, was placed above the model to keep track of energy flow. 

### 4.1. Effect of Grating Period on the Light Extraction Efficiency of the GaN Surface

**Figure 4:**
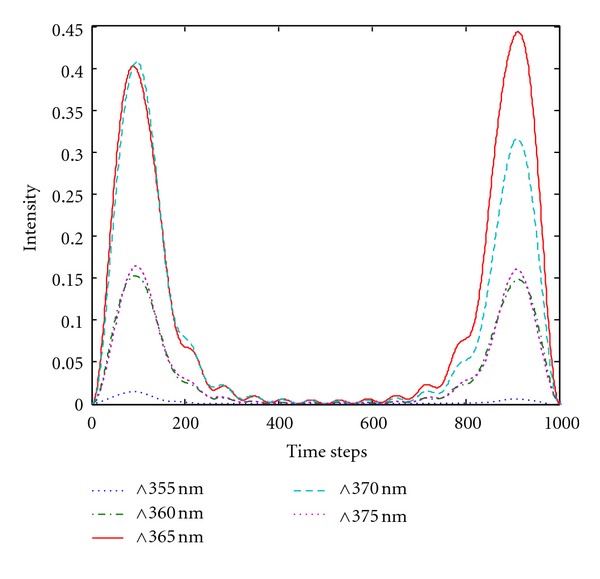
Energy flow of GaN at different grating periods.

**Figure 5:**
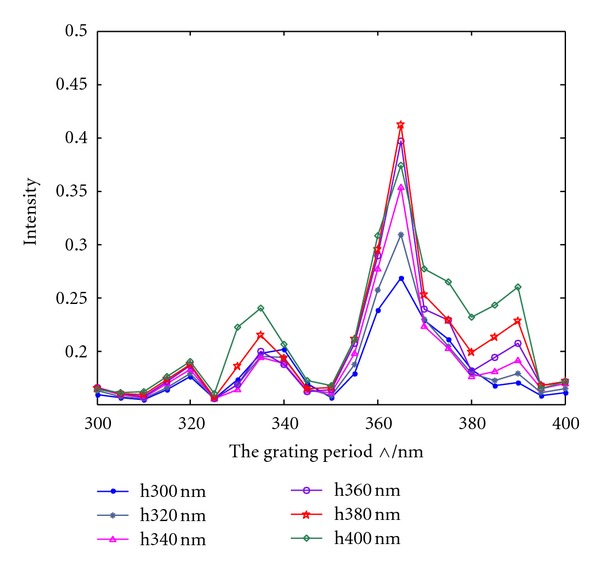
Relationship between the grating period of GaN and light extraction efficiency with silver film thickness ranging from 300 nm to 400 nm.

Figure 4 presents the energy flow of the GaN with a double-grating displacement structure at different grating periods. The maximum flow intensity value was 0.44, which can be obtained at a GaN surface grating period of 365 nm.  Figure 5 shows the relationship between the surface grating period of GaN and light extraction efficiency when the silver film thickness changes from 300 nm to 400 nm. For light extraction efficiency with oscillatory changes in grating period, the maximum value acquired constantly ranged from 360 nm to 380 nm. This result confirms that grating period influences the light extraction efficiency of LEDs. At a silver film thickness of 300 nm to 400 nm, the relationship curves of grating period and light extraction efficiency are similar and the maximum light extraction efficiency occurred at a 365 nm grating period. When the grating period continued to increase, the light extraction efficiency gradually declined. This result is attributed to two factors. First, the surface grating period is related to the wave vector that causes plasmon resonance. Thus, only when the grating period satisfies the excitation conditions can the SPs be excited. Consequently, visible light is extracted and transmissible light is enhanced. Second, the ideal size of diffraction grating satisfies the wavelength of light on a metal surface when the period is considerably smaller than the wavelength of light. Otherwise, conductivity to light diffraction is low and the effect of changing light directions caused by light diffraction decreases. On the other hand, when the period is far from the wavelength of light, the plane of the grating section is excessively large, thereby causing stronger total internal reflection, which slows down light emission.

### 4.2. Effect of Silver Film Thickness on Light Extraction Efficiency

**Figure 6:**
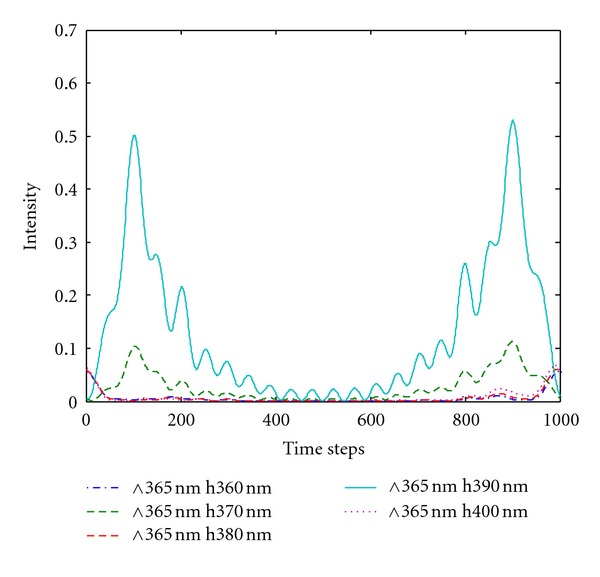
Energy flow of silver film (of different thicknesses) with a double-grating structure.

**Figure 7:**
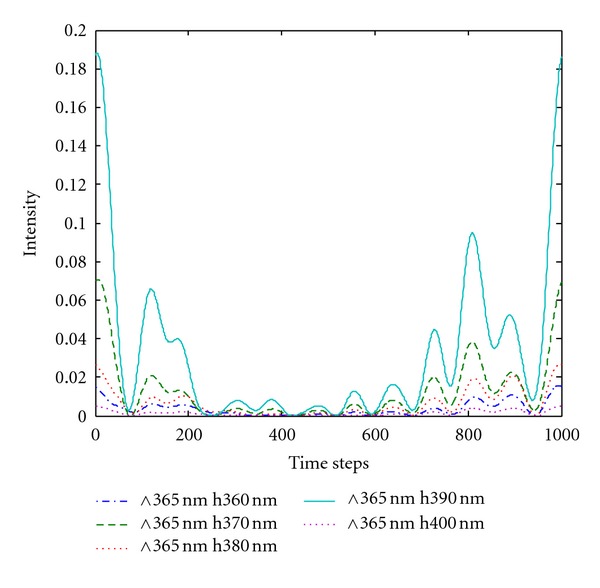
Energy flow of silver film (of different thicknesses) with a single grating structure.

The grating period was set at 365 nm on the basis of the analysis in Section 4.1. The thickness of the metallic silver film was set at 300 nm to 400 nm. Figures 6 and 7 show the energy flow of the silver film with a double-grating displacement structure and that of the silver film with a single grating, respectively. The light extraction efficiency of LEDs with a double-grating displacement structure improved by 43% over that of the silver film surface with a single grating. The graph shows that the silver film thickness should satisfy the grating coupling conditions to enhance light extraction efficiency. A silver film that is too thick or too thin cannot induce coupling. The plasmon resonance mode existed on both sides of the metal/dielectric material interface. Thus, when the film thickness was at the nanometer scale, the evanescent field formed by the SPs was strong enough to pass through the other side of the metal. Consequently, the SP electromagnetic fields on both sides of the metal film interacted with each other and formed a group of coupled SPs, as illustrated by the energy flow curve of silver thickness at 390 nm in Figure 6.

The plasmon resonance frequency reached the same value and energy flow intensity reached a maximum of 0.55. However, when the silver film thickness was smaller than 390 nm, the SP resonance frequency remained at the degenerate state, in which the energy flow intensity exhibited no significant enhancement. The results indicate the absence of a coupled phenomenon. With increasing silver film thickness, the energy flux rate gradually decreased, indicating the continuous division of the SP resonance frequency. Consequently, coupling disappeared when the silver film thickness tended toward infinity.

## 5. Conclusion

Grating-patterned etching was performed on GaN and silver film surfaces to establish an LED model with a double-grating displacement structure. The FDTD method was used in conducting a numerical simulation. A grating etched on the GaN surface effectively extracts a limited amount of light and enhances the light extraction efficiency of LEDs. At a GaN grating period of 365 nm and a silver film thickness of 390 nm, light extraction efficiency reaches a maximum of 55%.
